# Virtual Reality-Based Therapy Can Enhance Balance and Muscular Endurance in Children and Adolescents with Down Syndrome: A Systematic Review with a Meta-Analysis

**DOI:** 10.3390/bioengineering11111112

**Published:** 2024-11-04

**Authors:** Marina Piñar-Lara, Irene Cortés-Pérez, Ángeles Díaz-Fernández, María de Alharilla Montilla-Ibáñez, Ana Sedeño-Vidal, Esteban Obrero-Gaitán

**Affiliations:** 1 CAIT APROMPSI, C/Clara Campoamor 8, 23470 Cazorla, Spain; mpl00021@red.ujaen.es; 2 Department of Health Sciences, University of Jaén, Campus Las Lagunillas s/n, 23071 Jaén, Spain; andiaz@ujaen.es (Á.D.-F.); asedeno@ujaen.es (A.S.-V.); eobrero@ujaen.es (E.O.-G.); 3 Otorhinolaryngology Service, University Hospital “City of Jaen”, 23007 Jaen, Spain; alharillamontilla@gmail.com

**Keywords:** down syndrome, virtual reality, video games, exergames, postural balance, muscular endurance, meta-analysis

## Abstract

Physical exercises are crucial for enhancing postural balance and muscle strength in children and adolescents with Down syndrome (DS). Virtual reality-based therapy (VRBT), which utilizes exergames, can favor performing physical activity. The aim of this review was to assess the effectiveness of VRBT in improving balance and muscular endurance in children and adolescents with DS. Since inception up to August 2024, we screened in PubMed Medline, SCOPUS, WOS, CINAHL, and PEDro studies that assessed the effectiveness of VRBT, compared to conventional therapy (CT) or usual care (UC), in improving balance and muscular endurance in DS. Methodological quality was assessed using the PEDro scale. Effect size in meta-analyses was calculated with the Cohen’s standardized mean difference (SMD) and its 95% confidence interval (95% CI). Nine studies providing data from 424 participants with good methodological quality (PEDro: 6.3 ± 1.2 points) were included. Our meta-analysis showed that VRBT is more effective than controls in increasing function (SMD = 1.22; 95% CI 0.64 to 1.81; *p* < 0.001), dynamic balance (SMD = −2.2; 95% CI −3.1 to −1.25; *p* < 0.001), and muscular endurance (SMD = 1.37; 95% CI 0.58 to 2.2; *p* < 0.001). This is the first meta-analysis to exclusively focus on children and adolescents with DS, demonstrating the effectiveness of VRBT in enhancing balance and muscular endurance.

## 1. Introduction

Down syndrome (DS) is the most prevalent genetic neurodevelopmental disorder in children, occurring in approximately 1 in 800 [1] or 1 in 1000–1100 births worldwide [2]. DS is caused by the presence of an extra copy of chromosome 21 (Hsa21; trisomy 21) in somatic cells, resulting from a process known as nondisjunction [3]. Individuals with DS exhibit various motor and mental developmental clinical manifestations [4,5] as well as other conditions such as congenital heart diseases, low physical endurance, sleep disorders, or orofacial impairments [6,7,8]. The most significant motor impairments in DS are related to joints and muscles, characterized by joint laxity due to hypotonia or muscle weakness (resulting from reduced muscle tone), which negatively impacts muscle strength [9,10]. This predisposes individuals with DS to alterations in postural balance in both static and dynamic conditions, hindering coordination and mobility during functional tasks [11]. Additionally, visual and hearing disorders, among others, can contribute to distorted balance in individuals with DS [12]. Several studies have reported that decreased postural balance is correlated with a high risk of falls [13], restricting participation in school and social community activities and reducing quality of life [14]. Currently, more than 200,000 individuals are living with DS [15], and according to the new Global Burden of Disease of DS, the years lived with disability are increasing [16] highlighting the importance of conducting novel interventions that improve motor and balance disorders to reduce disability.

Given the critical role of visual, vestibular, hearing, and proprioceptive inputs in maintaining balance under both static and dynamic conditions [17], it is essential that therapies for these individuals incorporate these stimuli. To date, physiotherapy interventions, such as kinesio taping in feet muscles, classical balance training exercises, muscle strengthening, neuromuscular stretching exercises, and more innovative approaches like dance interventions or dual-task exercises, have demonstrated effectiveness in improving balance in these children [18,19,20,21]. In addition to them, virtual reality (VR) devices are novel technological devices that can be used in the rehabilitation of a wide variety of physical, cognitive, and psychologist pediatric conditions [22,23,24,25]. Virtual reality-based therapy (VRBT) is the use of VR devices for therapeutics purposes. VR is an advanced type of human–computer interface that allows users to immerse themselves in and engage with a highly realistic computer-generated environment, providing visual, auditory, and proprioceptive stimuli [26]. According to the level of immersion and presence offered to users with these VR devices, the most frequent modalities of VR are non-immersive or immersive. Non-immersive VR consists of the visualization of virtual environments in classical and bi-dimensional screens. In these systems, the individual can interact with the virtual environments using a mouse, hand-controller, or joystick [27]. Commercial examples of this technology are Nintendo or Xbox adding Kinect Sensor. Opposite, immersive VR, to date less used in research and clinical practice, favor the visualization of environments in 360° (full immersion) using head-mounted displays [28]. Meta Quest 2 and 3 is one of the most used immersive VR devices in rehabilitation. Exergames are the primary video games utilized in rehabilitation with VR devices. Particularly for children, the motivation derived from playing in these virtual and playful environments enhances participation in therapy, potentially increasing the benefits of VRBT [29]. VR devices enable the simulation and practice (including at home) of various sports, such as tennis, basketball, and football. Moreover, affordable commercial video games that promote repetitive physical exercise tasks tailored to each child’s specific disabilities can aim to activate and reorganize brain areas, catalyzing neuroplasticity changes related to improvements in movement, balance, or other cognitive functions [30,31].

Compared to others, neurodevelopmental disabilities more extensively studied in the literature, such as cerebral palsy [32], the use of exergames as VRBT in children and adolescents is a novel neurorehabilitation intervention that could potentially improve motor and balance impairments as well as physical condition in these individuals [33,34]. Several reviews published between 2015 and 2021 [35,36,37,38,39] have suggested the beneficial effects of VRBT, compared to conventional therapies (CT), in improving motor learning, balance, muscle strength, or aerobic endurance. While these reviews present insightful findings, recent studies published since the last review highlight the need to update the existing literature. To the best of our knowledge and to enhance the generalization of findings, standardizing the study population, including only studies that provide data on children and adolescents, is essential due to the fact that the majority of published research focuses on individuals within these age groups. Therefore, we hypothesize that VRBT could be effective in increasing balance and muscular endurance in this population. Therefore, the aim of our systematic review with a meta-analysis was to retrieve all published evidence to assess the effectiveness of VRBT in improving balance and muscular endurance in children and adolescents with DS. Additionally, we want to evaluate the effect of VRBT according to specific comparison therapies and whether the effect is similar in children or adolescents.

## 2. Materials and Methods

### 2.1. Type of Study and Register

To achieve the objective, a systematic review with meta-analysis was conducted following the Preferred Reporting Items for Systematic Reviews and Meta-analyses (PRISMA 2020 version) [40]. Additionally, the Cochrane Handbook for Systematic Reviews of Interventions and The Handbook of Research Synthesis and Meta-analysis were consulted for methodological aspects [41,42]. The quality of this systematic review was assessed using the AMSTAR 2 checklist [43]. Finally, the protocol of this systematic review was previously registered in PROSPERO database (CRD42024542230).

### 2.2. Literature Search and Databases Consulted

Since inception up to August 2024, two authors (M.P.-L. and I.C.-P.) conducted a literature search in PubMed Medline, SCOPUS, Web of Science (WOS), CINAHL Complete, and PEDro (Physiotherapy Evidence Database), which was supervised by an expert third author (E.O.-G.). Additionally, this search was complemented screening in the reference list of reviews previously published, proceedings, abstracts, and other sources such as Google Scholar that report studies from journals without impact factor. The research question of this review, according to PICOS tool [44], was as follows: Is VRBT more effective than others in increasing postural balance and muscular endurance in children and adolescents with DS? With the aim to retrieve the major quantity of potential studies to be included in the review, our search strategy was designed using the population (DS) and intervention (VRBT) conditions. It allows us to revise more studies, identifying more that meet the inclusion criteria. Therefore, the key terms used in our search strategy, according to MeSH Thesaurus, were “down syndrome”, “virtual reality”, “virtual reality-exposure therapy”, and “exergaming”. Additionally, other entry terms were used. Table 1 shows the search strategy used in each database and the search terms. Boolean operators (“AND” to join conditions, and “OR” to join related terms in each condition) and specific tags for each database were used. Finally, filters related to language, publication date, and free full access were not applied.

### 2.3. Study Selection: Inclusion and Exclusion Criteria

All studies retrieved with the literature search were revised in detail for two authors independently (M.P.-L. and I.C.-P.). When one author highlighted a study as having potential to be included in the review, it was examined by the two authors. The level of agreement between authors was known when calculating the Cohen’s kappa coefficient (κ) [45] (κ < 0, non-existent; 0 ≤ κ ≤ 0.2, non-significant; 0.2 < κ ≤ 0.4, discrete; 0.4 < κ ≤ 0.6, moderate; 0.6 < κ ≤ 0.8, substantial; and 0.8 < κ ≤ 1, excellent [46]. Finally, a third author (E.O.-G.) was consulted to solve doubts and discrepancies. The main inclusion criteria were established according PICOS tool: (1) population, children, and adolescents with DS; (2) intervention (VRBT); (3) control intervention (conventional therapy or usual care [UC]; (4) outcomes, postural balance, and muscular endurance; and (5) study design, randomized controlled trials [RCTs], pilot RCT or quasi-experimental studies with randomization, and two groups. Other inclusion criteria related to meta-analysis were that studies included must provide statistical data to conduct the meta-analysis. Oppositely, we excluded studies that included adults with DS and studies that compared two groups; one of them comprised healthy controls or studies that mixed participants with DS and other neurological diseases.

### 2.4. Data Extraction

Two authors (M.P.-L. and A.D.-F.), independently, were in charge of extracting the followings data from the studies included in the review: (1) overall characteristics of the studies: authors’ names, year of publication, setting, funding, type of study, blinding, total sample size, age and sex, and number of groups; (2) information of VRBT: modality of VR devices employed (non-immersive or immersive), VR device and video game used, and protocol of application in VRBT (number of sessions, weeks, sessions per week, and duration of each session); (3) information of control intervention: whether it was CT or UC and its protocol of application; and (4) data about variables of interest: name of each variable, test employed, and mean and standard deviation of the post-intervention assessment. In each case where standard deviation was not provided, it was estimated trough other data, such as interquartile range, standard error, or range, according to standardized and validated procedures [41,47]. Doubts in this stage were consulted with a third author (I.C.-P.).

### 2.5. Variables

The variables assessed in this systematic review were postural balance (functional, dynamic, and static) and muscular endurance. On the one hand, related to postural balance, functional balance is a postural balance’s dimension that informs the level of balance during functional tasks or activities; dynamic balance is the ability to maintain standing and stable during movements or displacements; and static balance is the ability to maintain the body in a position without displacements or movements [48,49,50]. On the other hand, muscular endurance refers to the capacity of muscles to sustain exercise [51].

### 2.6. Assessment of the Methodological Quality, Risk of Bias, and Quality of Evidence

The assessment of the methodological quality and the risk of bias in individual studies and quality of evidence of findings in each meta-analysis were performed by two authors independently (M.A.M.-I. and A.S.-V.) and discrepancies were solved by a third author (I.C.-P.).

The PEDro scale was used to assess the methodological quality, which has been recommended to use in physiotherapy RCTs [52]. This scale comprises 11 items that can be scored as yes (adding 1 point if criterion is met) or no (opposite) [53]. The total score obtained for each RCT can range from 0 to 10 by adding points 2 to 11. According to this, the methodological quality of each RCT can be excellent (10-9 points), good (8-6 points), moderate (5-4 points), and low (3-0 points) [54]. Using this scale can help us to identify biases if some criteria are not met. Items 2 and 3 are related to selection bias, items 5 and 6 with performance bias, and item 7 with detection bias.

The quality of evidence of each meta-analysis was estimated in basis of the Grading of Recommendations Assessment, Development, and Evaluation (GRADE) tool [55,56] and from Meader’s GRADE checklist [57]. To establish the quality or level of evidence, the items taken into account are risk of bias in individual studies, inconsistency (or heterogeneity), imprecision, and indirect evidence. The quality of evidence will be downgraded for each item not met and risk of publication bias. The quality of evidence can be strong (all items are met and results are consistent), moderate (one item is not met, and new studies can change the result of the meta-analysis), low (indicates instability of the findings), and very low (findings can be taken into account with a lot of caution).

### 2.7. Statistical Analysis

The statistical software Comprehensive Meta-Analysis version 4 (Biostat, Englewood, NY, USA) was used to perform the meta-analysis [58]. Meta-analyses and subgroup analyses only were conducted if at least 2 studies provided data for it [41]. The pooled effect size was calculated with the Cohen’s standardized mean difference (SMD) and its 95% confidence interval (95% CI) in a random-effects model for continuous data [59,60] graphically displayed in forest plots [61]. Pooled effect size was interpreted according Kinney et al. (2020), who suggested that the effect size in rehabilitation studies could be null (SMD 0), small (SMD 0.08–0.15), medium (SMD 0.19–0.36), and big (SMD > 0.4) [62]. When it was possible, as an additional analysis, we estimated the mean difference as effect result to compare it with the minimum clinically important difference (MCID) in outcomes assessed with the same measurement [63]. The literature suggests that to compare the mean difference with the MID value for a measurement test, the most important optimal method is used to assess the clinical relevance or importance of a result [64]. Risk of publication bias was assessed with the funnel plot, with the *p*-value for Egger test, and with the trim-and-fill method. Heterogeneity (inconsistency) was estimated with the chi-square test and its *p*-value (*p* < 0.05 confirms heterogeneity) and the degree of inconsistency of Higgins (I^2^) [65]. Heterogeneity can be null (I^2^ 0%), low (I^2^ 10–25%), medium (I^2^ 25–50%), or large (I^2^ > 50%) [66].

### 2.8. Secondary Analyses

As secondary statistical analysis, we conducted the following. First, a sensitivity analysis using the leave-one-out method (one study removed method) was performed with the aim to assess the contribution of each study in the global pooled effect size. Later, we conducted two subgroup analyses to assess the possible differences between the effect of VRBT according to the comparison intervention (VRBT vs. CT and VRBT vs. UC) and in function of the age group of the participants (children and adolescents). To assess if differences were statistically significant between group, a meta-regression was carried out.

## 3. Results

### 3.1. Study Selection

**Figure 1:**
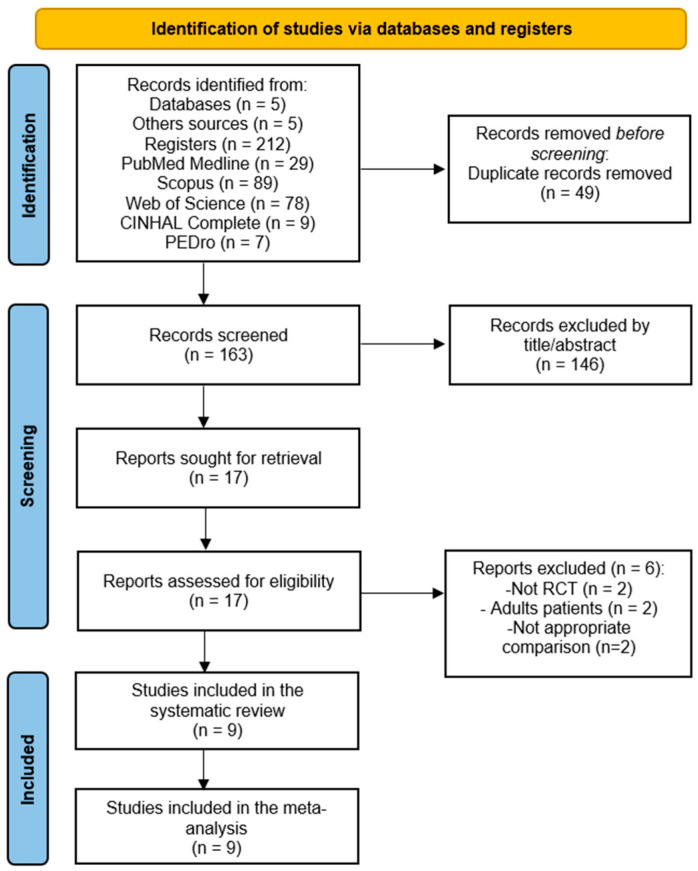
The PRISMA flow diagram for the study selection process.

The literature search retrieved 217 studies (212 from the five databases consulted and 5 from other sources). After removing duplicates, 163 were screened by title and abstract, of which 146 were excluded for not being relevant and 6 for not meeting the inclusion criteria. Two studies, although they met the inclusion criteria and were included in previous reviews, were excluded for providing data on adult individuals with DS [67,68]. Finally, nine studies were included in the present systematic review with a meta-analysis [69,70,71,72,73,74,75,76,77] (Figure 1). In the study selection process, an excellent level of agreement between authors was reported (κ = 0.89).

### 3.2. Characteristics of the Studies Included

The included RCTs, conducted in Taiwan [76], Indonesia [74], Saudi Arabia [69], Pakistan [70], Egypt [71], Brazil [72], Spain [77], and Chile [75] between 2010 and 2024, provided data from 424 individuals with DS (56% boys) with a mean age of 10.9 ± 3.1 years old. The inclusion criteria about the level of intellectual disability in the participants should allow us at least to understand the instructions of video games (mild–moderate intellectual disability). The intervention group who carried our VRBT comprised 188 participants (mean age of 10.7 ± 3.2 years old). Non-immersive VR devices were used as VRBT in eight RCTs [69,70,71,72,73,75,76,77] (Nintendo Wii video games being the most employed), and only one used immersive VR [74]. VRBT was used as unique therapy in all studies except for the study of Rahman et al., 2010, that was applied in combination with CT exercises [71]. The protocol application of VRBT in the included RCTs ranged from 8 to 60 sessions received during a period of 4 to 24 weeks between two and six sessions per week and between 20 and 60 min of VRBT exposition in each session. The control group comprised 236 participants (mean age of 10.6 ± 2.9 years old) received as control intervention traditional physical therapy [69,70,71,73,77] and UC [72,73,74,75,76].

According to outcomes measures, data for functional balance assessment were provided by six studies [69,70,71,72,73,74] from the Pediatric Balance Scale (PBS) and of the Bruininks–Oseretsky Test of Motor Proficiency 2’s (BOT-2); dynamic balance was assessed with data from the Timed-Up and Go Test (TUG) [69,74,77]; and static balance was assessed with posturographic parameters [70,75]. Finally, muscular endurance involved studies providing data from the 30 s Chair Stand Test (30-SCST), the Five-times Sit-to-Stand test (5-TSS), and the BOT-2 (strength dimension). All measurements informed about immediate post-intervention.

Finally, none of the included RCTs received external funding to perform the research. Table 2 shows the characteristics of the studies included in the meta-analysis.

### 3.3. Methodological Quality and Risk of Bias Assessments

The mean methodological quality of the studies included was good, showing a mean score in the PEDro scale of 6.3 *±* 1.2 points. Eight studies (89% of all) presented good methodological quality [69,70,72,73,74,75,76,77] and only one moderate [71]. The mean risk of bias in these studies was medium, and selection, performance, and detection were the most reported biases. Selection bias was identified in seven studies (77.7%) due to an inadequate concealed allocation (item 3 not met) [70,71,72,73,74,75,76]; performance bias was present in all studies due to the fact that participants and therapists could not be blinded (items 5 and 6 not met); and finally, detection bias was showed in five studies (55.5%) because evaluators were not blinded (item 7 not met) [69,70,71,72,75]. Table 3 showed in detail the PEDro assessment and bias reported for each study included.

### 3.4. Meta-Analyses

The studies included in the review provided statistical data to perform four meta-analyses (three for balance variables and another for muscular endurance). The findings of each meta-analysis have been summarized in Table 4.

#### 3.4.1. Functional Balance

**Figure 2:**
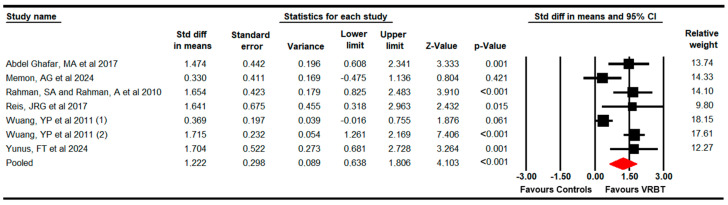
The forest plot of the effectiveness of VRBT in increasing functional balance [69,70,71,72,73,74].

The effectiveness of VRBT on functional balance was assessed, including six studies with seven independent comparisons [69,70,71,72,73,74] that provided data from 319 subjects with DS (31.7 per comparison). Our meta-analysis reported low-quality evidence of a large effect (SMD = 1.22; 95% CI 0.64 to 1.81; *p* < 0.001) favoring VRBT (Figure 2) without heterogeneity (I^2^ = 0%; Q = 4.2; df = 6; *p* = 0.38) or risk of publication bias (Egger *p* = 0.38 in Figure S1). Additionally, our findings showed an increase of 6.4 points (95% CI 1.6 to 11.12; *p* = 0.009) in the PBS compared to the controls. No variations were found using sensitivity analyses.

In subgroup analyses, on the one hand, meta-regression did not reveal differences between comparison therapies (*p* = 0.61) (Figure S2) and between children and adolescents (Figure S3).

#### 3.4.2. Dynamic Balance

**Figure 3:**
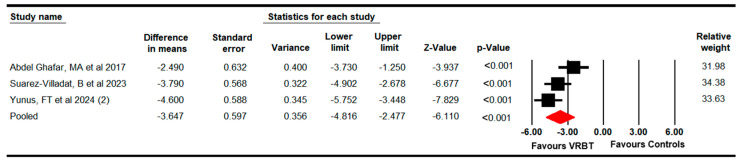
The forest plot of the effectiveness of VRBT in increasing dynamic balance [69,74,77].

Three studies with three independent comparisons [69,74,77] provided data from 95 participants with DS (31.7 per study) regarding the effectiveness of VRBT on dynamic balance. Our meta-analysis showed with very low-quality evidence that a large effect (SMD = −2.2; 95% CI −3.1 to −1.25; *p* < 0.001) favors VRBT (Figure 3) in increasing dynamic balance with moderate heterogeneity (I^2^ = 27.1%; Q = 2.7; df = 2; *p* = 0.3) and without risk of publication bias (Egger *p* = 0.42 in Figure S4). The meta-analysis showed that VRBT is able to reduce by 3.65 s (95% CI −4.81 to −2.48; *p* < 0.001) the performance of the TUG in comparison to the control intervention. No substantial differences were found after the sensitivity analysis.

#### 3.4.3. Static Balance (Posturographic Postural Control)

**Figure 4:**
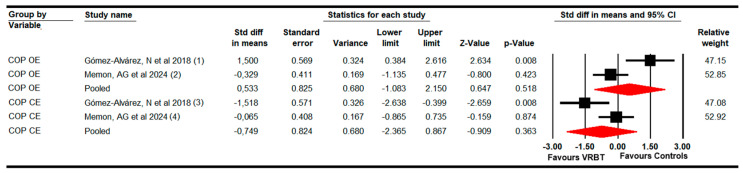
The forest plot of the effectiveness of VRBT in increasing static balance with open and closed eyes [70,75].

The ability to maintain the static balance after VRBT was assessed, including two studies with two independent comparisons [70,75] that provided data from 38 participants (19 per study) per eye condition. Our meta-analysis did not report statistically significant differences between VRBT and the controls in improving static balance in OE (SMD = 0.53; 95% CI −1.1 to 2.2; *p* = 0.52; I^2^ = 18.5%; Q = 1.2; df = 1; *p* = 0.3) and CE (SMD = −0.75; 95% CI −2.37 to 0.87; *p* = 0.36; I^2^ = 0%; Q = 0.7; df = 1; *p* = 0.4) (Figure 4).

#### 3.4.4. Muscular Endurance

**Figure 5:**
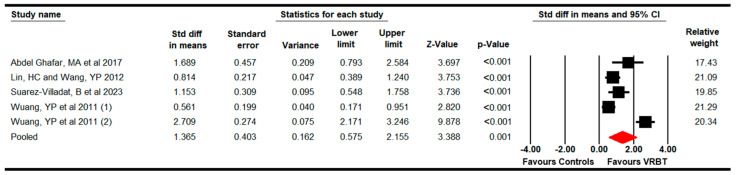
The forest plot of the effectiveness of VRBT in increasing muscular endurance [69,73,76,77].

The effect of VRBT on muscular endurance was assessed, including four studies with five independent comparisons that provide data from 314 participants (62.8 per study) [69,73,76,77]. The meta-analysis showed low-quality evidence of a large effect (SMD = 1.37; 95% CI 0.58 to 2.2; *p* < 0.001) favoring VRBT in increasing muscular endurance (Figure 5) without heterogeneity (I^2^ = 0%; Q = 3.7; df = 4; *p* = 0.4) or risk of publication bias (Egger *p* = 0.38 in Figure S5). The sensitivity analysis did not show variation in the pooled effect.

In subgroup analyses, according to specific comparisons, in increasing muscular endurance, VRBT was better than UC (SMD = 1.8; 95% CI 0.41 to 3.1; *p* < 0.001) but not than CT (SMD = 1.1; 95% CI −0.02 to 2.22; *p* = 0.053). Additionally, the meta-regression (*p* = 0.23) did not show differences between children and adolescents (Figure S6).

## 4. Discussion

Children and adolescents with Down syndrome often exhibit balance and muscle strength deficits [78]. VRBT has shown promise in addressing these impairments [79]. Previous reviews, including those by Lopes et al. (2020), included only two studies [37]; Stander et al. (2021), and Alba-Rueda et al. (2022), who included in their systematic reviews children, adolescents, and adults with DS, suggested that VRBT is a potentially effective therapeutic intervention for this population [38,39]. In addition, the only meta-analysis published to date included studies published up to 2021 [39]. However, recent studies published since 2021 and the need for a more homogeneous intervention group, including only children and adolescents with DS, prompted us to update the literature search and conduct a new meta-analysis. Previous reviews, including those by Lopes et al. (2020), Stander et al. (2021), and Alba-Rueda et al. (2022), have suggested VRBT as a potentially effective therapeutic intervention for this population. However, recent studies published since 2021 and the need for a more homogeneous intervention group prompted us to update the literature search and conduct a new meta-analysis. Therefore, the primary objective of this meta-analysis was to evaluate the efficacy of VRBT in improving balance and muscular endurance in children and adolescents with DS. Secondary objectives included comparing VRBT’s effectiveness to UC or CT and examining potential differences in outcomes between children and adolescents.

Following an updated literature search, we identified three additional studies published in 2023 [77] and 2024 [70,74] that met our inclusion criteria. Finally, our meta-analysis included nine studies providing data from 476 individuals, all children and adolescents with DS. Compared to previous reviews, the quality of evidence and generalizability of our meta-analysis were significantly enhanced due to the increased number of studies and participants. Furthermore, the sample was more homogenous after excluding two studies that included adults with DS [67,68]. Overall, our meta-analysis demonstrated that VRBT is an effective therapy for improving balance and muscular endurance in this population.

The primary outcome evaluated in this meta-analysis was the effectiveness of VRBT in enhancing balance, focusing on three specific dimensions: functional, dynamic, and posturographic static balance. The meta-analyses revealed that VRBT is highly effective in increasing functional and dynamic balance in comparison to CT or UC interventions. An additional analysis confirmed the clinical relevance of these improvements, with VRBT leading to a significant increase of 6.4 points in the PBS [69,70,72,74] and a reduction of 3.65 s in the TUG test [69,74,77]. While the MCID for the PBS and TUG has not been specifically calculated for children with DS, these results exceed the MCID values reported in similar populations for the PBS (5.83 points) and TUG (2.01 s) [80,81]. Both results are in line with the meta-analysis of Alba-Rueda et al. (2022), who showed that VRBT is better than other interventions (CT or UC) in improving functional and dynamic balance in patients of all age groups [39]. However, our meta-analysis differs in including two studies recently published for functional balance [70,74] and another for dynamic balance [77], and it excludes adults with DS and other studies that really did not provide data from functional [75] and dynamic balance assessed with the TUG [72,73,75,76] as they do. Subgroup analyses further confirmed the effectiveness of VRBT over the control or usual care interventions regardless of age (children or adolescents), highlighting its potential for both groups. Regarding static balance, VRBT was not better than others in improving postural control assessed with CoP excursion and Romberg’s test. This result may be attributed to the limited number of studies included in this analysis [70,75]. Further research with additional studies is needed to clarify the effects of VRBT on static balance in this population.

The final meta-analysis of our review demonstrated that VRBT increases muscular endurance in these patients without notable differences between children and adolescents. This suggests that VRBT can be an effective therapy for improving muscular endurance in these age groups. These results align with the meta-analysis by Alba-Rueda et al. (2022), although our meta-analysis included two studies more [69,77], excluding adults with DS [67,68]. A noteworthy finding from our subgroup analysis is that VRBT was superior to UC but not to CT. This implies that in clinical practice, physiotherapists may benefit from combining strength exercises using CT with VRBT to enhance dynamic balance.

The significant improvements observed in balance and muscular endurance can be attributed to several factors. First, all participants involved carried out physical exercises using video games, which have a large ludic component. These video games are named exergames, defined as video games that require body movements to play and function as a form of physical activity [82,83]. The gamification and immersive, ludic, and virtual environments of exergames are key advantages of VRBT, as they increase motivation and adherence to therapy [84]. The continuous challenges offered by these games also enhance curiosity, leading to greater engagement and physical activity, which can improve muscular endurance and balance. While 90% of the VRBT interventions used by the participants were non-immersive, primarily using the Nintendo Wii, these devices are characterized by their ease of use and comprehension, making them suitable for children with specific neurological conditions like DS or cerebral palsy [85,86]. Exergames such as Wii Sports, in which participants play tennis, golf, baseball, sky, soccer, or jumping, require global movements oriented toward functional tasks in the standing position. The repetitive practice of these movements can enhance muscular endurance. Another advantage of a VR device is that it provides somatosensory information (vestibular, auditory, visual, and proprioceptive) essential for maintaining and recovering balance [87]. The low postural control observed in these individuals, often due to hypotonia, muscle weakness and fatigue, and joint laxity, can reduce the gross motor skills and proprioceptive necessary for balance maintenance [74]. VR can address these challenges by inducing neuroplasticity. The multi-sensory inputs received by the brain during VR experiences can lead to neural changes that improve adaptive responses within the central nervous system [88]. Another advantage of VR devices is that they allow for customized therapies focused on patients receiving specific sensory stimuli related to the specific disability to recover from it (global movements, strength, balance, or cognitive tasks) [89]. Finally, the use of non-immersive VR devices, which are typically affordable and easy to use, allows individuals to perform these exercises at home. With proper instruction, parents or caregivers can supervise the activities carried out by children or adolescents [90].

To accurately interpret and generalize our findings, some limitations must be acknowledged. First, the relatively small number of studies included for certain variables and the limited sample sizes of individual studies reduce the quality of evidence, precision, and generalizability of our findings according to GRADE criteria. However, these studies represent the only published research that met our inclusion criteria, demonstrating the effectiveness of our search strategy. Another limitation is the medium risk of bias in some of the studies included, and selection, performance, and detection were the most reported biases that can affect the quality of evidence, accuracy, and generalization of our findings [91,92]. Regarding publication bias, it could not be assessed in meta-analyses with two or fewer studies due to limitations of the statistical software used. It is important to declare the impossibility of assessing if the effectiveness of VRBT will be sustained over time (follow-up). Ninety percent of the studies included used non-immersive VR devices, and these findings can be more supported by the use of non-immersive VR that require a lower level of complexity in its use. Finally, we were unable to assess the quality of evidence regarding the combined effect of CT and VRBT on improving balance in these children. We encourage researchers to perform future studies controlling the possible biases involved with a major sample size and using immersive VR devices and to assess if the effectiveness on balance and muscular endurance is major when CT approaches and VRBT protocols are applied together.

## 5. Conclusions

This meta-analysis, the first to focus exclusively on children and adolescents with DS, reveals that VRBT is effective in improving balance, particularly functional and dynamic balance, and muscular endurance in this population. While caution is warranted due to the limited number of studies included for certain variables, VRBT emerges as an effective and safe therapeutic option. The engaging and motivating nature of video games used to facilitate physical exercises enhances its appeal. Future research with larger sample sizes and the incorporation of immersive VR devices is necessary to solidify these findings.

## Figures and Tables

**Table 1:** Search strategy applied for each database.

Databases	Search Strategy
PubMed Medline	(down syndrome[mh] or down* syndrome[tiab] or syndrome, down [tiab] or trisomy-21[tiab]) AND (virtual reality[mh] or virtual reality[tiab] or reality, virtual[tiab] or virtual reality exposure therapy[mh] or virtual reality exposure therap*[tiab] or virtual reality immersion therap* [tiab] or reality therapy, virtual [tiab] or exergaming[mh] or exergam*[tiab] or virtual reality exercise[tiab] or active-video gam*[tiab])
SCOPUS	TITLE-ABS-KEY(“down syndrome” or “syndrome, down” or “trisomy-21”) AND TITLE-ABS-KEY(“virtual reality” or “reality, virtual” or “virtual reality exposure therapy” or “virtual reality immersion therapy” or “reality therapy, virtual” or “exergaming” or “exergame” or “exergames” or “virtual reality exercise” or “active-video game”)
Web of Science	TOPIC (*down syndrome* or *syndrome, down* or *trisomy-21*) AND TOPIC (*virtual reality* or *reality, virtual* or *virtual reality exposure therapy* or *virtual reality immersion therapy* or *reality therapy, virtual* or *exergaming* or *exergame* or *exergames* or *virtual reality exercise* or *active-video game*)
CINAHL Complete	AB (down syndrome or down, syndrome or trisomia 21) AND AB (virtual reality or reality, virtual or virtual reality exposure therapy or virtual reality immersion therapy or reality therapy, virtual or exergaming or exergame or exergames or virtual reality exercise or active-video game)
PEDro	Down syndrome and virtual reality

**Table 2:** Characteristics of the studies included in the meta-analysis.

Study	VRBT Group			Control Group		Variable (Test)	Qualitative Findings in Individual Studies	
	Sample Characteristics	VRBT Intervention Characteristics		Sample Characteristics	Control Intervention Characteristics		Intra-Group Differences	Inter-Group Differences
		VR System	Duration of VRBT					
Abdel Ghafar, MA et al., 2017 [69] (Saudi Arabia) Non-single blinded RCT Setting: Special Education School Funding: NR	13 children; 7.2 ± 1.9 years; Sex per group ND ID severity: IQ mild (SBIS)	NIVR device: Nintendo Wii Fit using sports video games.	24 sessions, during 8 weeks, 3 per week and 30 min per session	13 children; 7.4 ± 1.3 years; Sex per group ND	Traditional physical therapy, including balance exercises in standing and sitting position	Functional balance (PBS)	NR	Statistically significant differences favors VRBT group (*p* = 0.046)
						Dynamic balance (TUG)	NR	Statistically significant differences favors VRBT group (*p* = 0.043)
						Muscular endurance (5-TSS)	NR	Statistically significant differences favors VRBT group (*p* = 0.027)
Gómez-Alvárez, N et al., 2018 [75] (Chile) Non-blinded RCT Setting: Special Education School Funding: No	9 children; 8.3 ± 2.1 years; Sex per group ND ID severity: IQ NR	NIVR device: Nintendo Wii Fit Balance Board using sports video games.	10 sessions, during 5 weeks, twice per week, and 20 min per session	7 children; 8.4 ± 1.6 years; Sex per group ND	Usual care daily routine	Static balance (posturograpy)	Statistically significant intragroup differences in VRBT for closed eyes assessment (*p* = 0.039)	Not statistically significant differences between groups in any open and closed eyes condition (*p* > 0.05)
Lin, HC and Wang, YP 2012 [76] (Taiwan) Single-blind RCTSetting: University Funding: NR	46 adolescents, 15.6 ± 3.6 years; 21B:25G ID severity: IQ 52.5 ± 11.7 (WISC-III)	NIVR device: Nintendo Wii sports video games.	18 sessions during 6 weeks, 3 sessions per week, and 20 min per session	46 adolescents; 14.9 ± 3.9 years; 22B:24G	Usual care daily routine	Muscular endurance (BOT-2)	NR	Statistically significant differences favors VRBT group (*p* = 0.02)
Memon, AG et al., 2024 [70] (Pakistan) Non-blinded RCT Setting: Rehabilitation Center Funding: No	12 children; 8.1 ± 0.8 years; Sex ND ID severity: IQ 62.5 ± 3.5 (SBIS)	NIVR device: Nintendo Wii Fit using video games.	24 sessions during 8 weeks, 3 sessions per week, and 30 min per session	12 children; 7.6 ± 0.9 years; Sex ND	Traditional physical therapy, including balance exercises in standing and sitting position	Functional balance (PBS)	Statistically significant intragroup differences in VRBT (*p* = 0.005) and control (*p* < 0.01) groups	Not statistically significant differences between groups (*p* = 0.38)
						Static balance (posturograpy)	Statistically significant intragroup differences in VRBT (*p* < 0.05) and control (*p* < 0.05) groups	Not statistically significant differences between groups (*p* > 0.05)
Rahman, SA et al., 2010 [71] (Egypt) Non-blinded RCT Setting: Community association Funding: NR	15 children; 10.9 ± 1.2 years; 6B:9G ID severity: IQ 36–67 (SBIS)	NIVR device: Nintendo Wii Fit Balance Board using three sports video games. Additionally, individuals were performing conventional physical therapy exercises.	36 sessions during 6 weeks, 6 sessions per week, and 60 min per session	15 children; 11.6 ± 0.4 years; 7B:8G	Traditional physical therapy program comprising strengthening, walking, and climbing stairs exercises	Functional balance (BOT-2)	Statistically significant intragroup differences in VRBT (*p* < 0.001) and control (*p* = 0.017) groups	Statistically significant differences favors VRBT group (*p* < 0.001)
Reis, JRG et al., 2017 [72] (Brazil) Non-blinded RCT Setting: Community association Funding: NR	7 children; 9 ± 2.5 years; Sex ND ID severity: IQ NR	NIVR device: Xbox 360 plus Kinect sensor using two video games (River Rush and Hall of ricochets)	16 sessions during 4 weeks, 4 sessions per week, and maximal 20 min per session	5 children; 8 ± 2.5 years; Sex ND	Usual care daily routine	Functional balance (PBS)	NR	Statistically significant differences favors VRBT group (*p* = 0.01)
Suarez-Villadat, B et al., 2023 [77] (Spain) Single-blind RCT Setting: Special Education School Funding: NR	24 adolescents; 14.1 ± 1.2 years; 14B:10G ID severity: mild–moderate	NIVR device: Nintendo Wii Fit using sports video games.	60 sessions during 20 weeks, 3 sessions per week, and 60 min per session	25 adolescents; 14.3 ± 1 years; 16B:9G	Traditional physical therapy program comprising motor and coordination exercises related motor skills	Dynamic balance (TUG)	Statistically significant intragroup differences in VRBT (*p* = 0.002)	Statistically significant differences favors VRBT group (*p* = 0.038)
						Muscular endurance (30-SCST)	Statistically significant intragroup differences in VRBT (*p* = 0.008)	Statistically significant differences favors VRBT group (*p* = 0.027)
Wuang, YP et al., 2011 [73] (Taiwan) Single-blind RCT Setting: University Funding: NR	52 children; 7–12 years old (range) Sex ND ID severity: IQ NR	NIVR device: Nintendo Wii Fit using sports video games.	48 sessions during 24 weeks, twice per week, and 60 min per session	53 children; 7–12 years old (range); Sex ND	Traditional physical therapy, including balance exercises	Functional balance (BOT-2)	NR	Not statistically significant differences between groups (*p* > 0.05)
				50 children; 7–12 years old (range); Sex ND	Usual care daily routine	Muscular endurance (BOT-2)	NR	Not statistically significant differences between groups (*p* > 0.05)
Yunus, FT et al., 2024 [74] (Indonesia) Single-blind RCT Setting: University Funding: No	10 adolescents; 12.9 ± 3.2 years; Sex per group ND ID severity: IQ score 55–69	IVR device: Head mounted displays SenMor’s VR.	8 sessions, during 4 weeks, twice per week, and 20 min per session	10 adolescents; 12.9 ± 3.2 years; Sex per group ND	Usual care daily routine	Functional balance (PBS)	Statistically significant intragroup differences in VRBT (*p* < 0.001)	Statistically significant differences favors VRBT group (*p* < 0.001)
						Dynamic balance (TUG)	Statistically significant intragroup differences in VRBT (*p* < 0.001)	Statistically significant differences favors VRBT group (*p* < 0.001)

Abbreviations: VRBT, virtual reality-based therapy; VR, virtual reality; WISC-III, Wechsler Intelligence Scale for Children—Third Edition; IQ, intelligent quotient; SBIS, Stanford Binet intelligence scale; ND, non-defined; NR, non-reported; NIVR, non-immersive virtual reality; IVR, immersive virtual reality; RCT, randomized controlled trial; B, boys; G, girls; PBS, Pediatric Balance Scale; BOT-2, Bruininks–Oseretsky Test of Motor Proficiency 2; TUG, Timed-Up and Go Test, 30-SCST, 30-s Chair Stand Test; 5-TSS, Five-times Sit-to-Stand test.

**Table 3:** The PEDro assessment and biases reported in the studies included.

Study	Items											Total	Quality	Biases
	1	2	3	4	5	6	7	8	9	10	11			
Abdel Ghafar, MA et al., 2017 [69]	Yes	Yes	Yes	Yes	No	No	No	Yes	Yes	Yes	Yes	7/10	Good	Performance and detection
Gómez-Alvárez, N et al., 2018 [75]	Yes	Yes	No	Yes	No	No	No	Yes	Yes	Yes	Yes	6/10	Good	Selection, performance, and detection
Lin, HC and Wuang, YP 2012 [76]	Yes	Yes	No	Yes	No	No	Yes	Yes	Yes	Yes	Yes	7/10	Good	Selection and performance
Memon, AG et al., 2024 [70]	Yes	Yes	No	Yes	No	No	No	Yes	Yes	Yes	Yes	6/10	Good	Selection, performance, and detection
Rahman, SA et al., 2010 * [71]	Yes	Yes	No	Yes	No	No	No	No	No	Yes	Yes	4/10	Moderate	Selection, performance, and detection
Reis, JRG et al., 2017 [72]	Yes	Yes	No	Yes	No	No	No	Yes	Yes	Yes	Yes	6/10	Good	Selection, performance, and detection
Suarez-Villadat, B et al., 2023 [77]	Yes	Yes	Yes	Yes	No	No	Yes	Yes	Yes	Yes	Yes	8/10	Good	Performance
Wuang, YP et al., 2011 * [73]	Yes	Yes	No	Yes	No	No	Yes	Yes	No	Yes	Yes	6/10	Good	Selection and performance
Yunus, FT et al., 2024 [74]	Yes	Yes	No	Yes	No	No	Yes	Yes	Yes	Yes	Yes	7/10	Good	Selection and performance

Abbreviations: 1: Eligibility criteria; 2: random allocation; 3: concealed allocation; 4: baseline comparability; 5: blind subjects; 6: blind therapists; 7: blind assessors: 8: measures of at least one key outcome were obtained from more than 85% of the subjects initially allocated to groups; 9: intention-to-treat analysis; 10: between-group comparisons; 11: point estimates and variability. Note: The eligibility criteria item does not contribute to the total score. Note: The score of the studies marked with * was confirmed in the PEDro database: https://pedro.org.au/spanish/ (accessed on 3–10 September 2024).

**Table 4:** Main findings in meta-analyses.

	Effect Size						Heterogeneity		PUBLICATION BIAS			Quality of Evidence (GRADE)					
Variables	K	N	N_s_	SMD	95% CI	*p*	Q (df)	I^2^ (*p*)	Funnel Plot (Egger *p*)	Trim and Fill		Risk of Bias	Incons	Indirect	Imprec	Pub Bias	Quality
										Adj SMD	% var						
Functional balance	7	319	45.6	1.22	0.64 to 1.81	<0.001	4.2 (6)	0% (0.65)	Sym (0.38)	1.22	0%	Medium	No	No	Yes	No	Low
Dynamic balance	3	95	31.7	−2.2	−3.1 to −1.25	<0.001	2.7 (2)	27.1% (0.3)	Sym (0.42)	−2.15	0%	Medium	Prob.	No	Yes	No	Very low
Static balance OE	2	38	19	0.53	−1.1 to 2.2	0.52	1.2 (1)	18.5% (0.3)	NP	NP	NP	Medium	Prob.	No	Yes	Prob.	Very low
Static balance CE	2	38	19	−0.75	−2.37 to 0.87	0.36	0.7 (1)	0% (0.4)	NP	NP	NP	Medium	No	No	Yes	Prob.	Very low
Muscular endurance	5	314	62.8	1.37	0.58 to 2.2	<0.001	3.7 (4)	0% (0.4)	Sym (0.24)	1.37	0%	Medium	No	No	Yes	No	Low

Abbreviations: K, number of comparisons; N, total sample size; Ns, participants per study; SMD, standardized mean difference; 95% CI. 95% confidence interval; *p*, *p*-value; Q, Q-test; df, degree of freedom; I^2^, degree of inconsistency; Adj, adjusted; % var, percentage of variation; RAGT, robotic-assisted gait training; TT, treadmill training; CT, conventional therapy; Funct. Indep, functional independence; NP, not possible to calculate.

## Data Availability

Data will be made available upon reasonable request to the corresponding author.

## Supplementary Materials

The following supporting information can be downloaded at https://www.mdpi.com/article/10.3390/bioengineering11111112/s1, Figure S1: Funnel plot for functional balance (overall); Figure S2: Scatterplot of SMD on therapy comparisons (functional balance); Figure S3: Scatterplot of SMD on age groups (functional balance); Figure S4: Funnel plot for dynamic balance (overall); Figure S5: Funnel plot for functional balance (overall); and Figure S6: Scatterplot of SMD on therapy comparisons (muscular endurance).
