# Supplementary material for: Virtual Reality-Based Therapy Can Enhance Balance and Muscular Endurance in Children and Adolescents with Down Syndrome: A Systematic Review with a Meta-Analysis
Source: Bioengineering (Basel). 2024 Nov 4;11(11):1112. doi: 10.3390/bioengineering11111112 (PMC11591943; doi:10.3390/bioengineering11111112)
Supplement: Supplementary file 1 [file bioengineering-11-01112-s001.zip › bioengineering-3251907-supplementary.pdf]

SUPPLEMENTARY FILE: SUPPLEMENTARY FIGURES

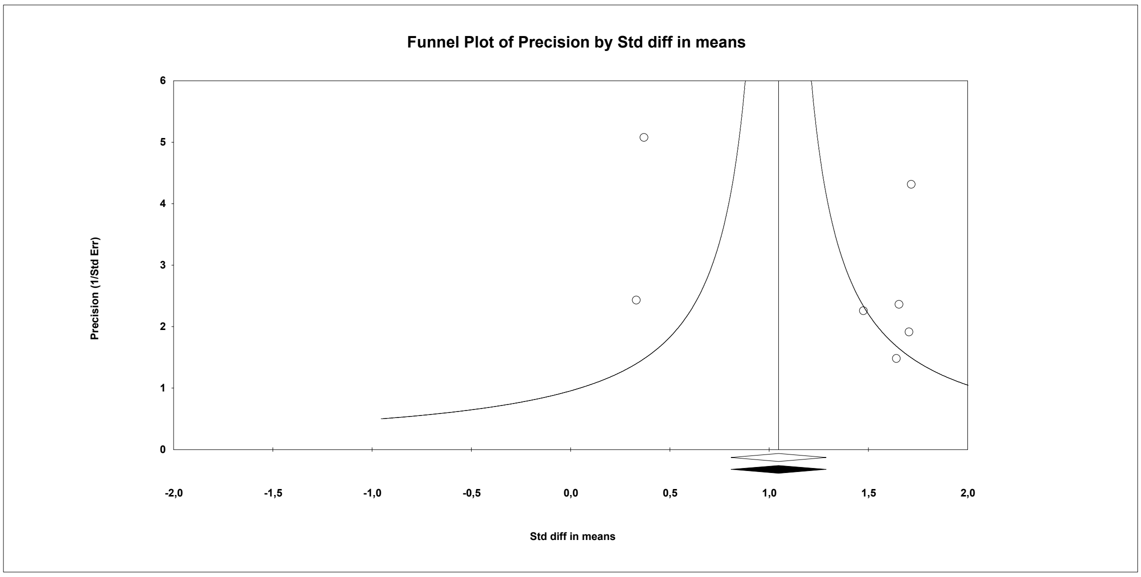

Figure S1: Funnel plot for functional balance (overall)

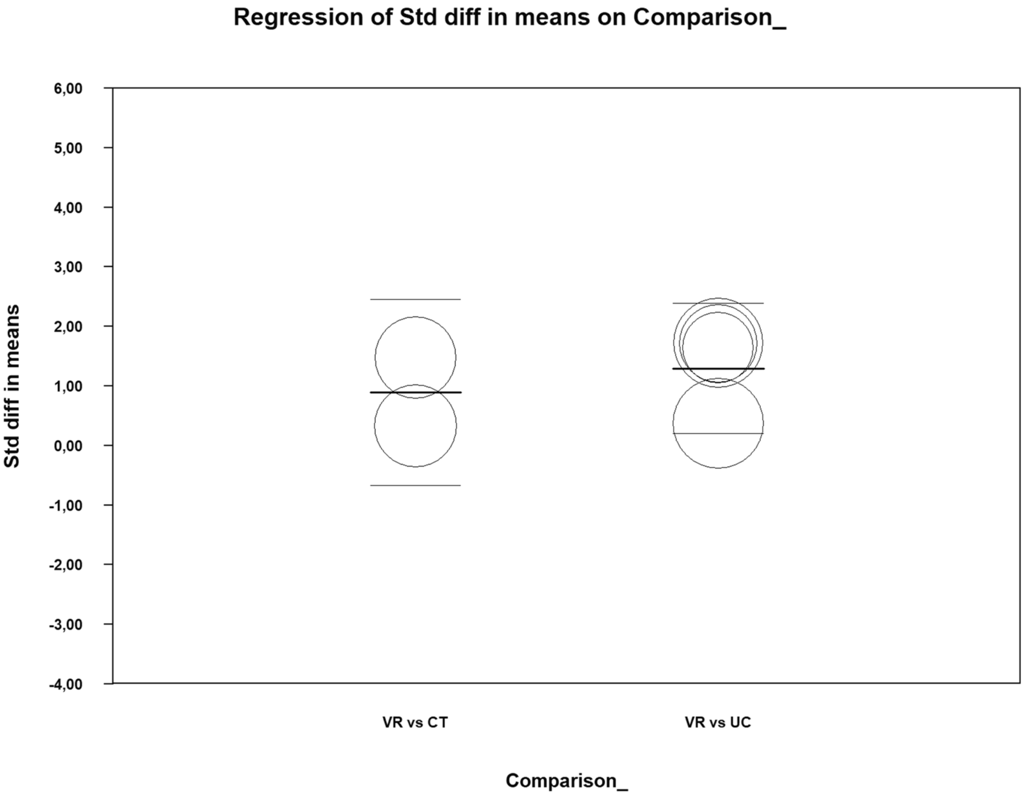

Figure S2: Scatterplot of SMD on therapy comparisons (functional balance)

SUPPLEMENTARY FILE: SUPPLEMENTARY FIGURES

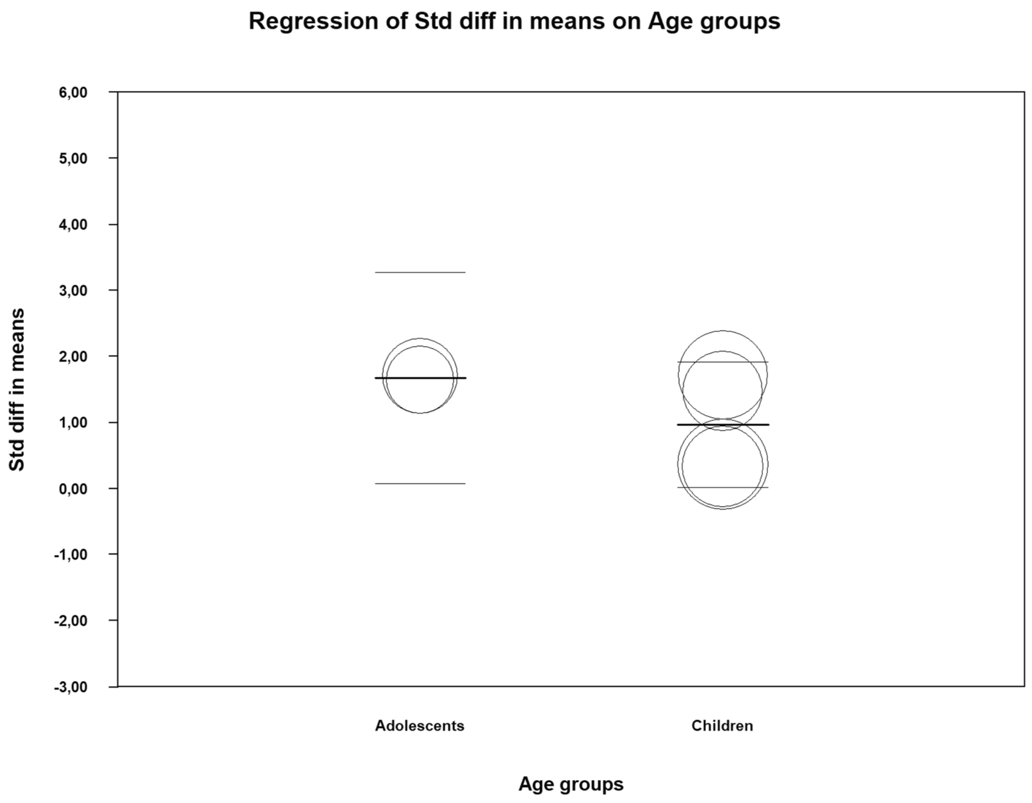

Figure S3: Scatterplot of SMD on age groups (functional balance)

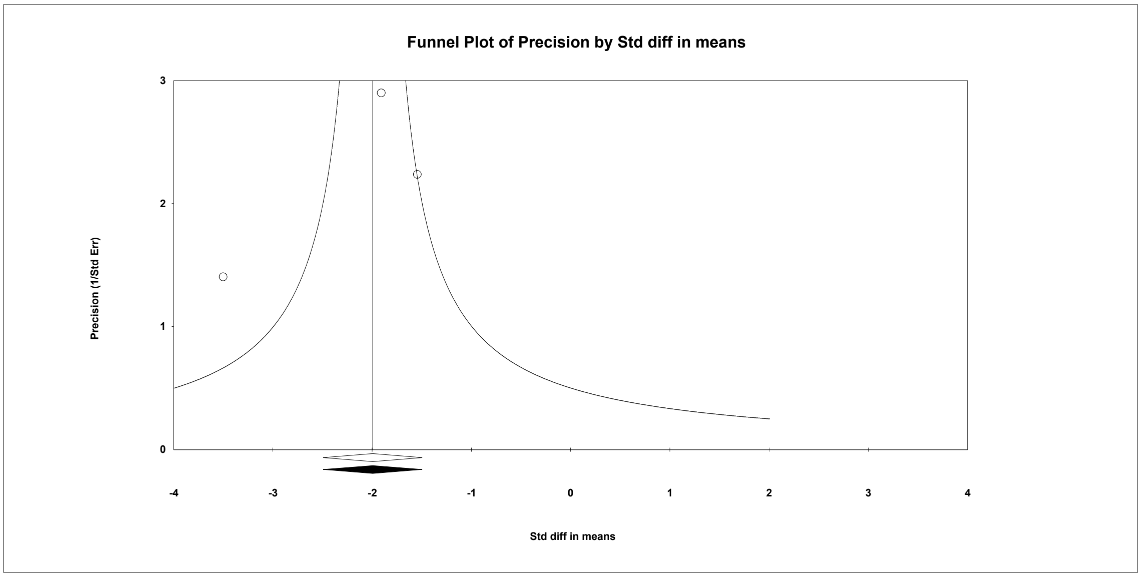

Figure S4: Funnel plot for dynamic balance (overall)

SUPPLEMENTARY FILE: SUPPLEMENTARY FIGURES

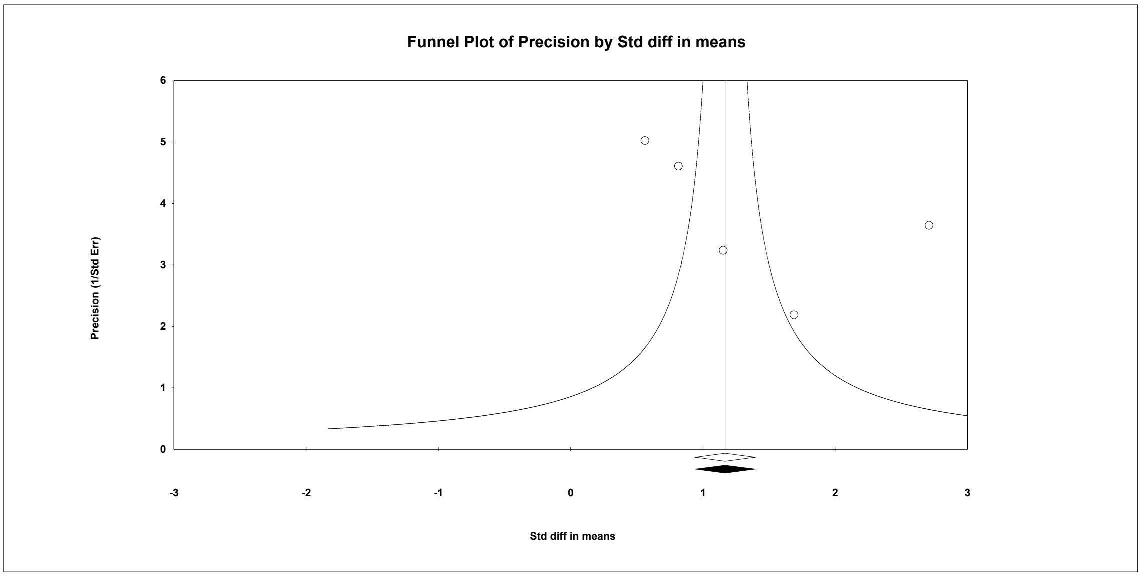

Figure S5: Funnel plot for functional balance (overall)

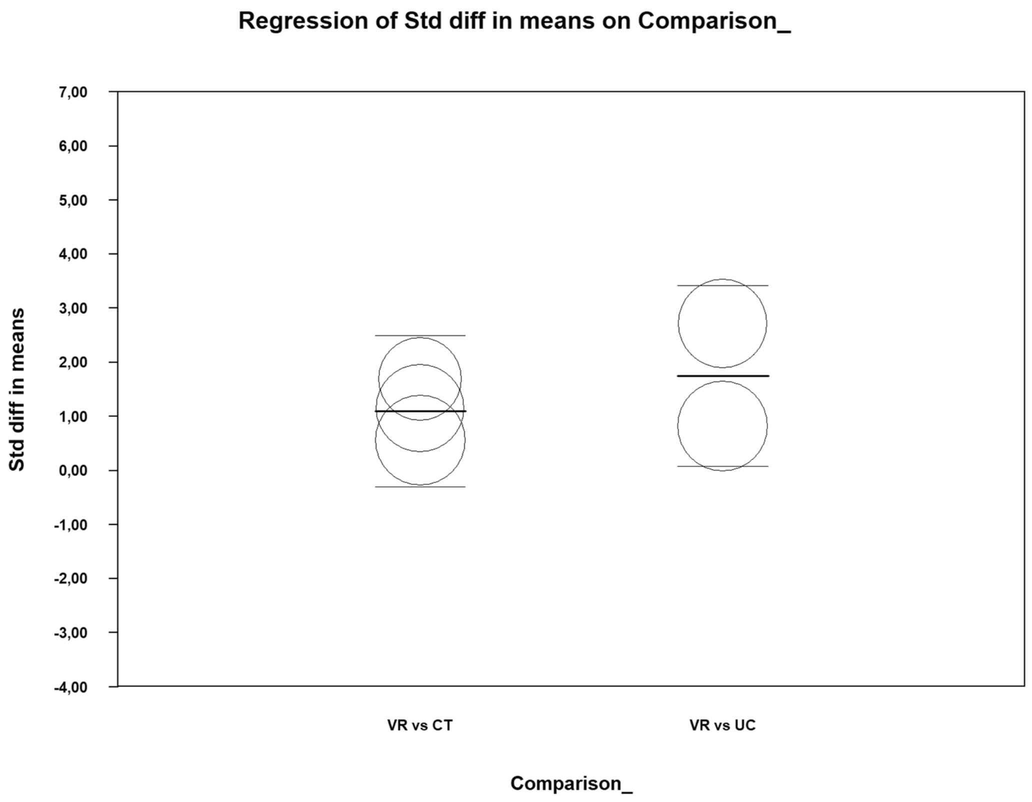

Figure S6: Scatterplot of SMD on therapy comparisons (muscular endurance)
